# Microcolonial Fungi on Rocks: A Life in Constant Drought?

**DOI:** 10.1007/s11046-012-9592-1

**Published:** 2012-10-17

**Authors:** Kristina Zakharova, Donatella Tesei, Gorji Marzban, Jan Dijksterhuis, Timon Wyatt, Katja Sterflinger

**Affiliations:** 1 Department of Biotechnology, University of Natural Resources and Life Sciences Vienna, Muthgasse 18, 1190 Vienna, Austria; 2 CBS Fungal Biodiversity Center, Uppsalalaan 8, 3584 CT Utrecht, The Netherlands

**Keywords:** Black yeast, Anhydrobiosis, Two-dimensional gel electrophoresis, Protein profiling, Trehalose

## Abstract

Black microcolonial fungi (MCF) and black yeasts are among the most stress-resistant eukaryotic organisms known on Earth. They mainly inhabit bare rock surfaces in hot and cold deserts of all regions of the Earth, but some of them have a close phylogenetic relation to human pathogenic black fungi which makes them important model organisms also with respect to clinical mycology. The environment of those fungi is especially characterized by extreme changes from humidity to long periods of desiccation and extreme temperature differences. A key to the understanding of MCF ecology is the question about metabolic activity versus dormancy in the natural environments. In this study, the time lag from the desiccated state to rehydration and full metabolic activity and growth was measured and defined in accordance with simulated environmental conditions. The ability to survive after desiccation and the speed of rehydration as well as changes of the whole cell protein pattern are demonstrated. Whereas both mesophilic strains—*Exophiala jeanselmei* and *Knufia perforans* (=*Coniosporium perforans*)—show a clear reaction toward desiccation by production of small proteins, *Cryomyces antarcticus*—the extremotolerant MCF—does not show any response to desiccation but seems just to down-regulate its metabolism. Data on intracellular sugar suggest that both trehalose and mannitol might play a cell protective role in those fungi.

**Electronic supplementary material:**

The online version of this article (doi:10.1007/s11046-012-9592-1) contains supplementary material, which is available to authorized users.

## Introduction

Black microcolonial fungi (MCF) and black yeasts together with lichens and cyanobacteria are among the most stress-tolerant organisms on the Earth [6]. They are found in the hot deserts of Arizona (USA) [24, 27], the cold Antarctic deserts [20, 22], as well as in Mediterranean countries, for example, Italy and Greece [10, 30]. Many of those rock inhabiting fungi—especially species of the genera *Exophiala* and *Knufia* (=*Coniosporium*)—cluster within the order *Chaetothyriales* and *Hysteriales* and thus have a close phylogenetic relation to human and animal pathogens like *Cladophialophora* spp., *Exophiala dermatitidis* or *Knufia epidermidis* (=*Coniosporium epidermidis*) [2, 34].

MCF form black, clump-like colonies consisting of isodiametrically dividing cells on rock surfaces, in cracks, pores and fissures of the rock and in micropits, created by their own deteriorative activity. Their morphology is interpreted as response to multiple stress factors: keeping the surface-volume ratio optimal decreases the loss of water and minimizes the colony surface with direct exposure to sun light and other physical and chemical stressors [28, 29, 31]. Recent experiments showed that the stress resistance of MCF against solar radiation, radioactivity, desiccation and oligotrophic conditions even allows them to survive space and Martian conditions. Therefore, black fungi are promising organisms for investigating the life in outer space and for gamma radiation experiments [23]. Humidity, temperature and solar radiation, deposition of organic/inorganic and nutrients on rock surfaces influence the settlement, growth and development of microorganisms. The stress resistance in those fungi might have played a significant role for the evolution of virulence factors in human pathogen species. Although other habitats may offer more extreme conditions of temperature, pH and salinity, they are rarely subjected to such rapid and extreme instability in physical and chemical conditions. It is known that on the rock surfaces, where microbial interactions occur, there are constant changes in atmospheric conditions. In this sense, as assumed by Gorbushina [11], ubiquitous subaerial biofilms are bioindicators that are continually subjected to climate change. Any changes in the composition of the atmosphere and climate are sensed by life on the rocks, which in turn affects atmospheric composition through its metabolic activity and biologically induced weathering.

The changing environmental conditions force rock inhabitants into periods of stress-induced dormancy, which are suddenly interrupted by the occasional return of growth-favorable conditions. Only organisms which have a very broad range of tolerance to multiply and fluctuating stress can survive under these harsh conditions. The term poikilo-tolerant (resistant to variable stress; from poikilos: variegated) has been used to describe the behavior of living organisms in environments where tolerance to multiply and variable parameters is essential for survival [11].

The real challenge to survive in the desert environments is desiccation, and neither bacteria nor archaea are specialists of survival under conditions of matrix stress. One of the driest and Mars-like environments on Earth—the area around the Yungay station of the hyperarid Atacama Desert (Chile)—is nearly free of any cultivable bacteria. Nevertheless, several hyphomycetes survive in this region in a re-cultivable state by the formation thick-walled spores [5]. That is why fungi are really survival specialists of complete desiccation by producing spores and also prosper—even though slowly—at extremely low levels of water availability [32].

Organisms, which are able to tolerate essentially complete dehydration, are known to be in anhydrobiosis; black rock inhabiting fungi can therefore also supposed to be anhydrobiotes. It is reported that anhydrobiotic organisms—during the process of desiccation—enter a state of metabolic arrest which is reversible on rehydration [13, 19].

As it is supposed by Sterflinger et al. [32], in the polar environment, it is well possible that the fungi are in dormant state most of the year and that they are only active during the short summer period when temperature rises and melting water is available. On the contrary, hot desert fungi might profit from dewfall that develops in deserts during a short time before sunrise [21]. This process would necessitate a very fast rehydration and up-regulation of the metabolic activity. For this reason, the aims of the study was: (1) to investigate how fast MCF can react to changes of humidity, (2) to examine under which conditions these fungi are active in their natural environments and (3) to analyze how they respond to dehydration and rehydration on the proteome and RNA level.

To answer these questions, selected strains of MCF were subjected to desiccation and rehydration in climate chambers and subsequently the protein pattern was analyzed by 2D gel electrophoresis. Protein expression profiles characterize states of dormancy, activity and growth related to different ecological conditions [14].

## Materials and Methods

### Model Organisms

The model organisms used for this study are as follows: (1) *Exophiala jeanselmei* MA 2853, a rock inhabiting black yeast closely related to opportunistic pathogens in humans; (2) *Knufia perforans* MA 1299, a mesophilic but highly stress-tolerant fungus found in hot and dry environments, like the Mediterranean, formerly named *Coniosporium perforans* [34]. Both, *E. jeanselmei* and *K. perforans,* can therefore also be addressed as extremotolerants, (3) *Cryomyces antarcticus* MA 5682, an extremophilic fungus from Antarctica. The strains were obtained from the ACBR culture collection (Austrian Center of Biological Resources and Applied Mycology, www.acbr-database.at).

### Rehydration Experiments

In nature, the fungal biomass is presented in such limited amount that it is impossible to perform any analysis. This study is a first attempt to find out the survival mechanisms of these fungi; therefore, the model system which reflects normal conditions was created.

All experiments were done in triplicate (2 biological and 3 technical replicates), and the average was taken. Inoculi were prepared as cell suspensions and drop-inoculated onto a sterilized cellophane membrane (Model 583 gel dryer Backing. Catalog# 1650963, Bio-Rad), which were placed on the surface of 2 % malt-extract agar (MEA, Applichem GmbH, Darmstadt, Germany). *E. jeanselmei* and *K. perforans* were grown at 20 °C and *C. antarcticus* at 15 °C for 30 days. Fresh biomass was harvested by scratching the material from the plates using a scalpel, then transferred to a sterile tube, and immediately frozen in liquid nitrogen and stored at −80 °C for further analysis.

In order to analyze the response to desiccation, the cellophane membranes with well grown fungal colonies were detached from the MEA and transferred into empty Petri dishes, which were subjected to dehydration in a climate chamber containing silica gel and dried to their constant weight which was reached after 6 days (this was tested in pre-experiments, see supplementary). The water loss, estimated by weight—measured using scales (Non-automatic weighing instrument ME235S-OCE, Sartorius mechatronics)—was about 90 % of fresh biomass, as a consequence of the severe water loss involved; this treatment was defined as desiccation.

Part of the dried biomass was immediately frozen in liquid nitrogen for the further analysis. Other cellophane membranes were replaced onto fresh MEA and immediately incubated in a climate chamber at 98 % rH. During the process of rehydration, samples were taken at 3 time points: after 3 min, 10 min and 1 h. After each sampling, the biomass was immediately frozen in liquid nitrogen for further experiments: (1) RNA extraction and quantification and (2) 2-D gel electrophoresis.

### RNA Extraction

Total RNA was extracted from frozen samples (150-200 mg) according to manufacturer’s guidelines (TRIzol reagent, Invitrogen). The RNA pellet was air-dried and then dissolved in distilled water (DNase/RNase Free; Invitrogen) during incubation for 10 min at 69 °C.

RNA quality and quantity was analyzed using a Nano Drop 1000 Spectrophotometer according to the manufacturer’s instruction.

### Protein Extraction and 2D Gel Protein Profiling

Extraction of whole cell protein and 2-D gel electrophoresis was carried out according to a protocol which was specially adapted for black fungi by Isola et al. [14]. The Bradford protein Assay [4] was performed to determine the concentration of proteins in fungal extracts. Reactions were carried out in microtiter plates according to the manufacturer’s instructions. A standard curve was established using serial dilutions from 0.8 to 100 μg ml^−1^ of bovine serum albumin (BSA). The resulting optical density (OD) at 595 nm was analyzed with a plate reader (Magellan; Tecan Austria, Grödig, Austria). All experiments were carried out in triplicate. For absolute amounts of RNA and proteins in fresh—non-desiccated—samples, the relative amounts of protein and RNA were calculated in relation to the dried biomass of the samples. The weight loss was quantified according to the weight change during desiccation. In the same way, the relative amounts in fresh biomass were calculated from absolute values in desiccated biomass. For each gel, 20 μg of protein was applied. IEF separation was performed using 13-cm strips pH 3-10NL. 2D gels were made in triplicate for each condition (fresh, dried, after 3 min and 1 h of re-hydration), the 3 gels were matched by warping (Image Master 2D Platinum version 5.0, Amersham Biosciences, Swiss Institute of Bioinformatics, Geneva, Switzerland) and the sum of all spots which were present at least in two gels was taken into account for the protein pattern analysis.

### Isolation of Sugars and HPLC Analysis

The dry and fresh mycelium samples (50–450 mg) were frozen in liquid nitrogen and placed in a stainless steel grinding jar (Qiagen, Venlo, The Netherlands. Catalog# 69985) and pulverized with the Qiagen Tissuelyser^®^ (2 min at 30 strokes/s). Before a second pulverization step (2 min at 30 strokes/s), 1–2 ml MQ was added. After the grinding, the sample was transferred into eppendorf tube and centrifuged (10,000×*g* at 4 °C, for 30 min). The supernatant was collected, heated for 30 min at 95 °C and centrifuged another 30 min at 10,000×*g*. Again, the supernatant was collected, filtered (Pall Life Science, Acrodisc Cr13 mm Syringe filter) and stored at −20 °C until used for sugar analysis by HPLC.

Detection and quantitative analysis of saccharides and polyols was carried out by HPLC [36] equipped with an IR detector (Waters, 2414 refractive index detector), a Ca+ cation-exchange column (Waters, SugarPak I column) and a mobile phase of MQ with 0,1 mM Ca EDTA (Sigma. Catalog# 340073). The column temperature was kept at 50 °C with a column heater (Waters), and a flow of 0.5 ml min^−1^ was maintained during separation (Waters, 515 HPLC pump). The autosampler (Waters, 717 plus autosampler) injected 10 μl per sample, and peak integration and calculation was performed with Empower software delivered. As standards trehalose, mannitol, glucose, glycerol, erythritol and arabitol were used.

## Results

**Fig. 1:**
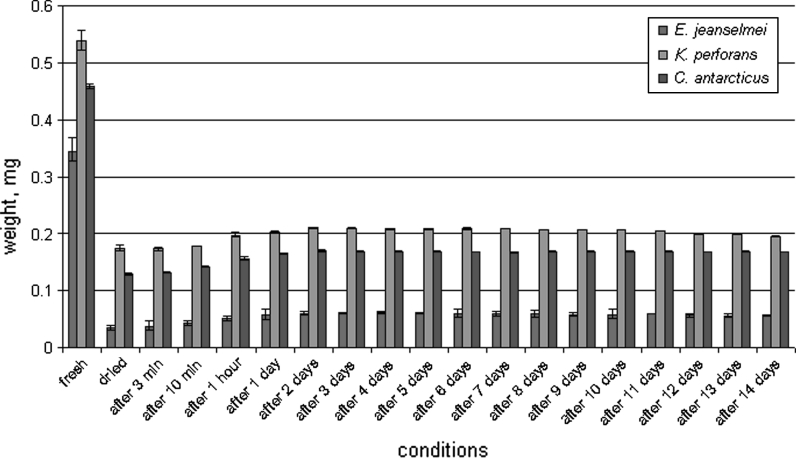
Rehydration of MCF and *black* yeast. Fresh biomass was subjected to the desiccation until the constant weight was reached (on 6th day), and then dried biomass was rehydrated for 14 days. The weight was estimated for all the samples at different conditions: starting from 3 min of rehydration till the 14th day of rehydration period

After a desiccation period of 6 days, the maximum water loss was reached in the three fungal strains tested: water loss was 90 % for *E. jeanselmei*, 70 % for *K. perforans* and 70 % for *C. antarcticus*. After rehydration, *E. jeanselmei* re-gained 14.8 %; *K. perforans,* 36.4 %; and *C. antarcticus,* 33.9 % of the weight within 1 h. However, after 1 day of rehydration, *E. jeanselmei* gained 16.6 %; *K. perforans,* 37.3 %; and *C. antarcticus,* 35.8 % of the original biomass weight and stopped at this level (Fig. 1). An additional experiment for 14 days, when dried biomass was replaced into empty Petri dishes and exposed to rehydration at 98 % rH, was carried out. The results showed that the fungi did not gain their original fresh weight again; however, all three strains were able to grow again, after transferring to fresh medium, and thus proved to be alive and active after desiccation and rehydration.

**Fig. 2:**
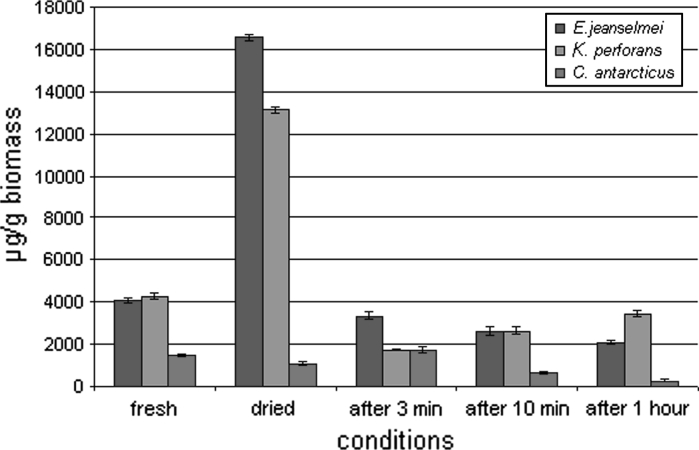
Absolute protein concentration of MCF and *black* yeast. Protein amount of the biomass was measured before and after desiccation, and also at different conditions: after 3 min, 10 min and 1 h of rehydration

**Fig. 3:**
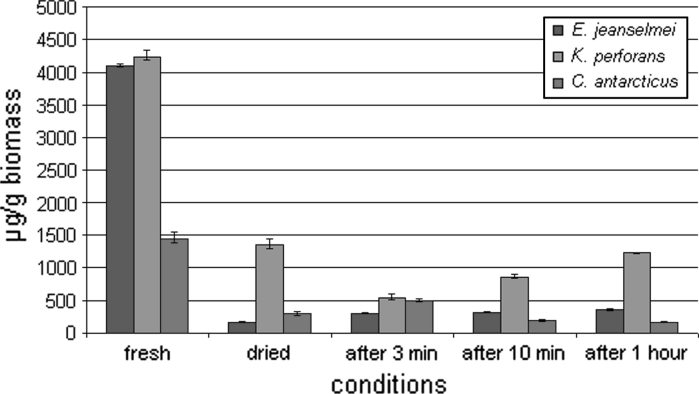
Relative protein concentration of MCF and *black* yeast. Protein content was re-calculated to the hydrated biomass according to the water loss of the samples

In *E. jeanselmei* and *K. perforans,* the amount of proteins in relation to the whole fungal biomass significantly increased upon drying due to the water loss within a period of 6 days (Fig. 2). The protein content increased from 4,000 to 16,800 μg g^−1^ for *E. jeanselmei* and from 4,100 to 13,200 μg g^−1^ for *K. perforans*. However, in *C. antarcticus,* the protein content decreases even in relation to the dry biomass from 1,700 to 1,500 μg g^−1^. The protein content of the fully hydrated cells was low: 4,000 μg g^−1^ for *E. jeanselmei*, 4,100 μg g^−1^ for *K. perforans* and 1,700 μg g^−1^ for *C. antarcticus* (Figs. 2, 3) and when protein values were calculated back to the fully hydrated state, it can be seen that the values did not show a significant increase during the rehydration period.

**Fig. 4:**
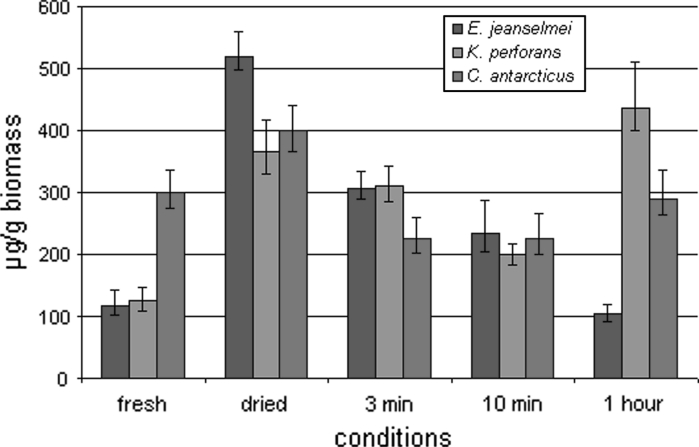
Absolute RNA concentration of MCF and *black* yeast. RNA concentration of the biomass was measured before and after desiccation and also at different conditions: after 3 min, 10 min and 1 h of rehydration

**Fig. 5:**
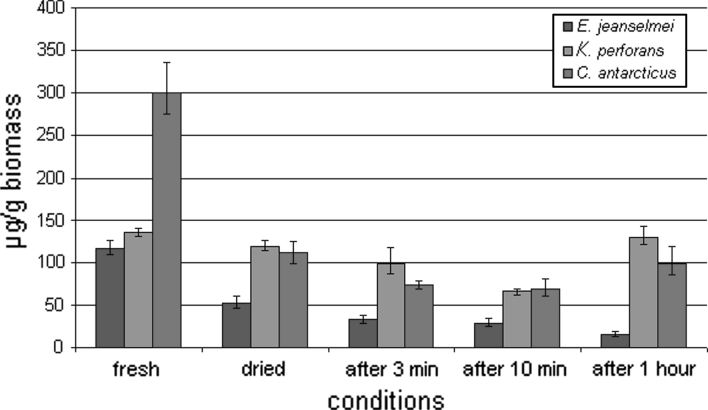
Relative RNA concentration of MCF and black yeast. RNA concentration was re-calculated to the hydrated biomass according to the water loss of the samples

**Fig. 6:**
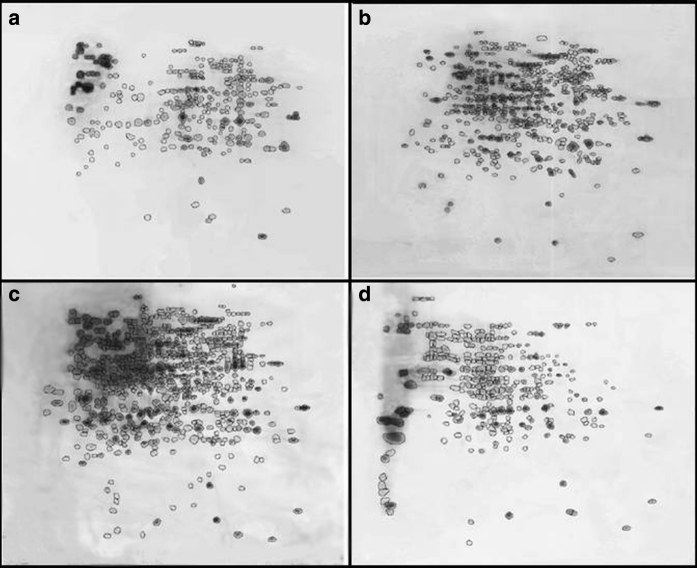
Comparison of 2D gel protein expression profiles of *K. perforans*: **a** fresh biomass, **b** after desiccation for 6 days, **c** after rehydration for 3 min and **d** after rehydration for 1 h. For each gel, 20 μg of protein was applied. IEF separation was performed using 13-cm strips pH 3-10NL

The absolute amount of RNA (Fig. 4) was lower in fully hydrated colonies—when grown on MEA—than in the fully desiccated biomass and also in the rehydrated biomass. In contrast, the relative amount of RNA (Fig. 5)—recalculated according to the loss of water—shows that the cell activity decreased during desiccation. In *E. jeanselmei,* the RNA concentration was decreasing within all measurements. In colonies grown on MEA, it was 117 μg g^−1^, after drying the concentration decreased to 52 μg g^−1^ and after 1 h of rehydration 16 μg g^−1^. However, the cells, after drying for 2 weeks—this additional experiment was mentioned before—and being inoculated onto MEA, showed the ability to grow. In contrast, in *K. perforans,* RNA concentration decreased from 125 μg g^−1^ in fresh biomass to 119 μg g^−1^ after drying (absolute and related to fresh biomass). The minimal concentration 66 μg g^−1^ was reached after 10 min of rehydration. Then, concentration increased and reached 159 μg g^−1^, thus indicating up-regulation of the metabolism after 10 min of rehydration. In *C. antarcticus,* the results demonstrated that RNA concentration decreased that 300 to 111 μg g^−1^ after desiccation, and the minimal concentration was 69 μg g^−1^ after 10 min of rehydration. After 1 h of rehydration, the concentration was increasing thus indicating up-regulation of the metabolism after 1 h of rehydration. The re-activation of the metabolism after 10 min in *K. perforans* and 1 h, respectively, in *C. antarcticus* is also indicated by the whole cell protein content and the changes of the protein patterns as follows:

In all fungi tested, the protein profile clearly changed during the process of desiccation and again during the process of rehydration both in the number of protein spots and in the protein pattern. During desiccation, *K. perforans* shows a significant increase in protein spots from 275 to 474. Especially, the number of proteins in the higher pH range and with lower molecular weight is increased (Fig. 6). While 262 spots were matching in the fresh biomass compared with the dried biomass, 212 new spots were induced. In addition to small proteins, also some spots with higher molecular weight were detected as reaction to dehydration. After 3 min of rehydration, an increase from 474 to 589 spots was detected, indicating that the cell already after this short time period reacts to the availability of water. After 1 h of rehydration, the amount of spots decreased to 323 spots. Since especially those spots that were expressed after desiccation disappeared again after 1 h of rehydration, it can be concluded that the status of the cells normalizes during this period.

**Fig. 7:**
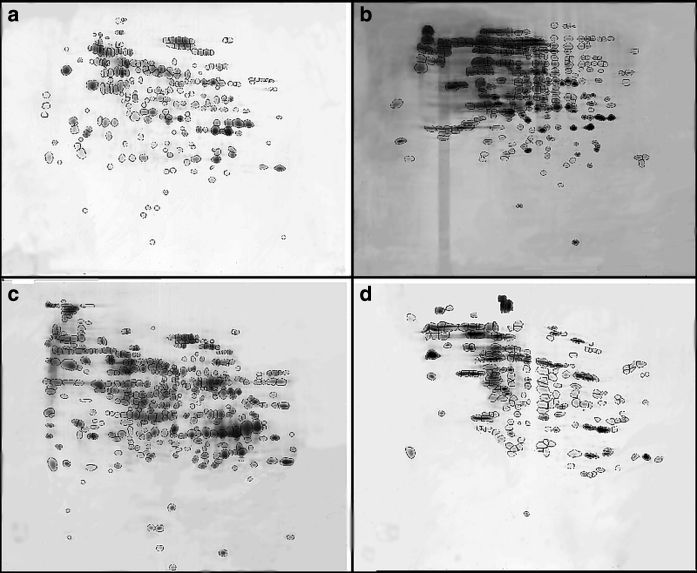
Comparison of 2D gel protein expression profiles of *E. jeanselmei*: **a** fresh biomass, **b** after desiccation for 6 days, **c** after rehydration for 3 min and **d** after rehydration for 1 h. For each gel, 20 μg of protein was applied. IEF separation was performed using 13-cm strips pH 3-10NL

In *E. jeanselmei,* there is a smaller increase in protein spots during desiccation as compared to *K. perforans*. Among the 280 spots shown after desiccation, 156 are pair with the 233 spots expressed in the fresh biomass (Fig. 7). However, also in this fungus, the protein pattern suggests that there is a strong response toward water loss since the pattern in the desiccated fungus differs in 122 spots from the fully hydrated colonies when grown on MEA. After 3 min in 98 % rH, a high number of proteins was detected, but after 1 h, the protein number is decreasing (showing 213 spots) and the protein pattern goes back to the level of the fresh biomass from MEA medium.

**Fig. 8:**
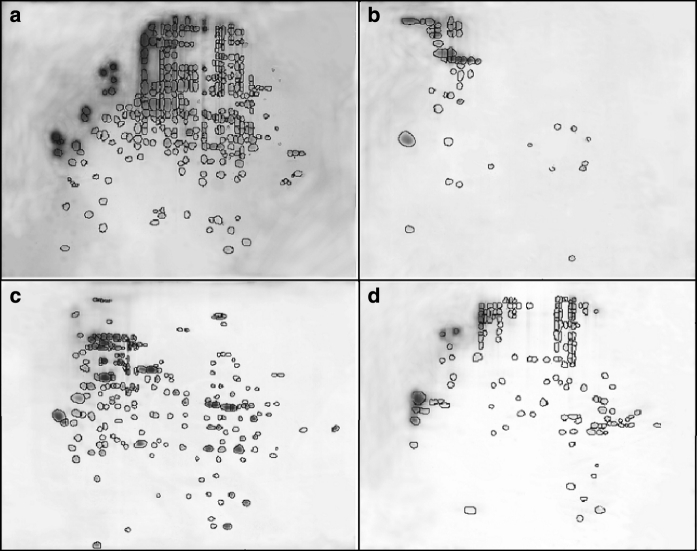
Comparison of 2D gel protein expression profiles of *C. antarcticus*: **a** fresh biomass, **b** after desiccation for 6 days, **c** after rehydration for 3 min and **d** after rehydration for 1 h rehydration for 1 h. For each gel, 20 μg of protein was applied. IEF separation was performed using 13-cm strips pH 3-10NL

In contrast to *K. perforans* and *E. jeanselmei, C. antarcticus* reacts to desiccation by reducing the number of protein spots from 323 in fresh biomass to 52 in the dried cells (Fig. 8). In the dried state, 34 big spots were exhibited.

**Fig. 9:**
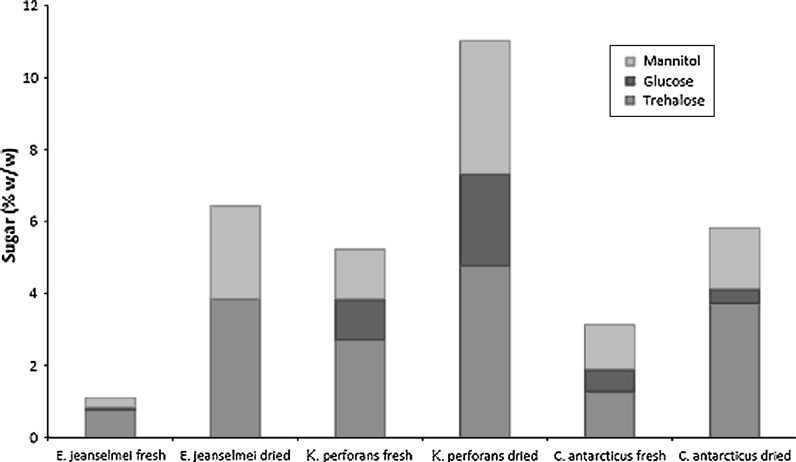
Sugars (% w/w) present in fresh (fully hydrated) biomass and dried biomass (after desiccation for 6 days). The amount of mannitol, glucose and trehalose estimated for each sample

Sugar analysis shows that trehalose and mannitol are the main solutes in the three fungal species of this study. Accumulation of the solutes is already prominent in wet samples and is increased in dried samples (Table 1; Fig. 9). The increase in the amount of sugars was most extensive with *E. jeanselmei*, which is corresponding with the amount of weight loss after drying of this fungus.

**Table 1:** Sugars (% w/w) present in fresh (fully hydrated) biomass and dried biomass after desiccation for 6 days

Species	Trehalos	Glucose	Mannitol	Total sugars
*E. jeanselmei* fresh	0.76	0.07	0.26	1.10
*E. jeanselmei* dried	3.85	0.00	2.59	6.44
*K. perforans* fresh	2.71	1.14	1.38	5.23
*K. perforans* dried	4.77	2.57	3.69	11.03
*C. antarcticus* fresh	1.28	0.62	1.24	3.13
*C. antarcticus* dried	3.73	0.41	1.68	5.83

## Discussion

Until now, the only factors that are known to be involved in stress adaptation of MCF are the strong melanisation of the multilayered cell walls, the microcolonial morphology, as well as the production of trehalose which helps to stabilize enzymes during periods of desiccation and the production of glycerol that is induced by osmotic stress [12, 29, 38]. The ability to survive in a desiccated state was shown to be an important feature to withstand periodical high temperatures that might occur on the rock in deserts and in the Mediterranean climate [29]. However, protein patterns and RNA levels as influenced by desiccation and rehydration were analyzed in this study for the first time.

The results of this study clearly show that the extremotolerant fungi *E. jeanselmei* and *K*. *perforan*s show a different response to desiccation and rehydration than the extremophilic fungus *C. antarcticus*. The fact that both MCF fungi (*K. perforans* and *C. antarcticus*) lost less weight than *E. jeanselmei* can be explained by the bigger portion of dry biomass in MCF which is mainly due to their multi-layered cell walls but also by the EPS layers of *E. jeanselmei,* which normally retains high amounts of water [29]. Thus, the absolute amount of water in MCF is lower than in the thin-walled cells and in the EPS of *E. jeanselmei*. It can also be concluded that the fully hydrated state may never be reached in nature—growth on laboratory medium and with levels higher than 90 % rH is artificial; those levels of nutrient availability and humidity are never reached in the natural environment of MCF.

The protein profiles and the RNA quantification shown in this study support the hypothesis that MCF and black yeasts are able to be active with low levels of intracellular water and are able to react to rehydration after periods of desiccation in a short time. The protein content of the wet cell is very low, which indicates massive amounts of other materials in the cell, as cell walls or solutes, which was shown by Sterflinger [28]. Trehalose and mannitol are both known to accumulate upon exposure to various types of stress and are the most abundant compatible solutes in conidia of Aspergillus [7, 8, 25, 29, 37]. Conidia are survival structures [17] and have medium resistance to heat, drought and other stressors [37]. The accumulation of these compounds is of the same order of magnitude as those of the conidia [18]. These data suggest that accumulation of these compounds is a normal aspect of vegetative growth of these fungi. The data of this study also show that the basic level of sugar in the fully hydrated cells is already very high, thus necessitating no induction of sugar production when desiccation is starting. The even higher amount of sugars inside of the dried biomass is rather due to the weight loss during desiccation than to a real increase in the sugars in sense of additional sugar production.

The protein patterns of *E. jeanselmei* and *K. perforans* indicate a modification of existing proteins and an expression of additional protective proteins during the process of desiccation. There is an obvious increase in large proteins suggesting the formation of clusters from protein plus chaperon. Again, the increase in the spot patterns after 3 min of rehydration in *K. perforans* can probably be interpreted as a release of the protective chaperons from the clustered proteins.

In contrast to *E. jeanselmei and K. perforans*, *C. antarcticus* did not show a significant change in the protein pattern but a dramatic decrease in the number of spots. This indicates that *C*. *antarcticus* rather down-regulates the metabolism than inducing a number of protective proteins. This would also mean that the proteins necessary for the survival of the colony are either desiccation resistant without any additional protective proteins or that other cellular components—for example, sugars or fatty acids—are involved in the protein protection. However, this fungus seems to have a slower reaction to rehydration than *K. perforans*. This can possibly be explained by the fact that *C. antarcticus* reacts to yearly cycles of anhydrobiosis and its activity is depending on the Antarctic seasons while *K. perforans* lives in the moderate and semi-dry environment with short cycles of dryness and humidity due to rain events and dew fall thus necessitating a faster response to rehydration. Also from the phylogenetic point of view, *Cryomyces* is highly distinct from the genera *Knufia* and *Exophiala*. While *Knufia* and *Exophiala* cluster within the order *Chaetothyriales* [34], *Cryomyces* forms a distinct clade without any obvious direct ancestor [26].

In general, desiccation together with anhydrobiosis—defined as a complete loss of “free” water from an organism—is an extreme stress [35]. Desiccation tolerance is characterized by the physical and chemical adjustments in order to withstand the dehydration and resume the biological activity after rehydration [15]. This process is a result of a complex cascade of molecular events, which can be divided into signal precipitation, signal transduction, gene activation and biochemical changes leading to the acquisition of desiccation tolerance. It is proposed that proteins related to desiccation tolerance and involved in the metabolic changes, protection against oxidation and other putative protective molecules show a particular abundance during the desiccation. Small heat-shock proteins (sHSPs), also called molecular chaperons, cluster with enzymes and stabilize their conformation [1, 16]. Late embryogenesis abundant (LEA) proteins are among the molecules with increased abundance during drying of plants [3, 15]. Another important process that has been described in moss—*Tortula ruralis*—is the packing of mRNA transcripts into mRNPs (messenger ribonucleic protein complexes) with polysomes that allows a rapid availability of these transcripts upon rehydration [9]. The results of this study give evidence that also the MCF and black yeast analyzed here have some general response to desiccation: The formation of small sized proteins in *K. perforans* and *E. jeanselmei* can possibly be interpreted as production of HSPs; bigger protein spots occurring after rehydration possibly are proteins the conformation of which is stabilized by molecular chaperons. However, there are two novel and important findings that will have to be studied in more detail in order to understand the ecology and systems biology of the MCF:

1. In *C. antarcticus,* there is an enormous loss of protein spots during desiccation indicating a down-regulation of the whole metabolism. The production of small proteins as reaction toward stress was not observed. Although the process of down-regulation—compared to production of HSPs—is less energy consuming and might reflect the oligotrophic environment of this fungus, it necessitates that the basic set of proteins is either highly specialized and thus not affected by desiccation or protected by other cell components which are not yet understood. Interestingly, Tesei et al. [33] demonstrated that *C. antarcticus* also does not produce any HSPs as a response to temperature stress. In order to understand the resistance of those proteins, identification by mass spectrometry will be carried out in the next study.
2. When rehydrated in 98 % rH, none of the fungi tested was able to gain the fully hydrated state as on MEA. However, the protein pattern suggests a normalization of the metabolism after approximately 1 hour. For this reason, it will be necessary to carry out studies on the content of cellular water and water retaining abilities of these fungi using either in situ colonies from fresh environmental samples or—since samples are often difficult to obtain and the available biomass is extremely low—further experiments should be carried out with dehydrated laboratory grown cultures.

From here, we can conclude that *E. jeanselmei* and *K. perforans*—both being extremotolerant but mesophilic—show a clear response to desiccation. There is strong evidence that they react by the production of proteins which can be interpreted as small HSPs and chaperons as well as large protein clusters formed by protein plus chaperon. In *C. antarcticus*—the extremophilic fungus from Antarctica—very few large spots—indicating cluster formation—were detected, and there was no indication of small HSPs production. The most prominent reaction to dehydration, namely the loss of protein spots, indicates a down-regulation of the metabolism and related proteins. All fungi show a remarkable fast response to water availability. Moreover, the protein patterns indicate that the fungi are able to gain a full metabolic activity in a state of low cellular water content. The results of this study give a first impression of cellular reactions toward anhydrobiosis in black fungi. Of course, the nature of the proteins will have to be identified in further studies based on mass spectrometry.

## Electronic supplementary material

Below is the link to the electronic supplementary material.

Supplementary material 1 (TIFF 916 kb)
