# Supplementary figures and images for: Microcolonial Fungi on Rocks: A Life in Constant Drought?
Source: Mycopathologia. 2012 Oct 17;175(5):537–47. doi: 10.1007/s11046-012-9592-1 (PMC3669513; doi:10.1007/s11046-012-9592-1)

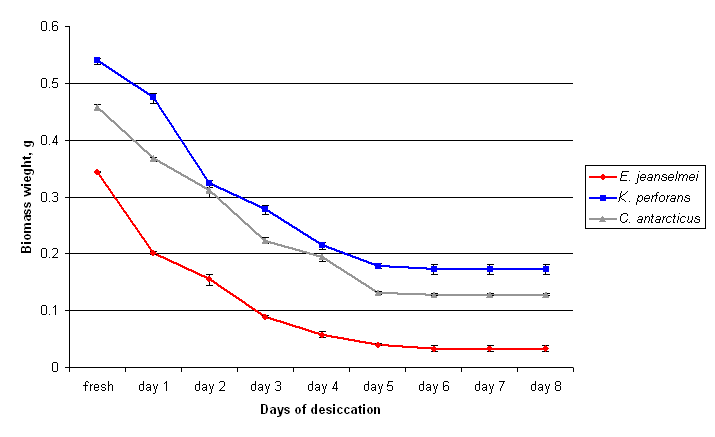

Supplement: Supplementary file 1 — Supplementary material 1 (TIFF 916 kb) [file 11046_2012_9592_MOESM1_ESM.tif]
